# A new method for modelling biological invasions from early spread data accounting for anthropogenic dispersal

**DOI:** 10.1371/journal.pone.0205591

**Published:** 2018-11-27

**Authors:** Luca Butikofer, Beatrix Jones, Roberto Sacchi, Marco Mangiacotti, Weihong Ji

**Affiliations:** 1 Institute of Natural and Mathematical Sciences, Massey University, Private Bag, North Shore Mail Centre, NZ-0745 Auckland, New Zealand; 2 Department of Earth and Environmental Sciences, University of Pavia, Via Taramelli, Pavia, Italy; Universidad de Sevilla, SPAIN

## Abstract

Biological invasions are one of the major causes of biodiversity loss worldwide. In spite of human aided (anthropogenic) dispersal being the key element in the spread of invasive species, no framework published so far accounts for its peculiar characteristics, such as very rapid dispersal and independence from the existing species distribution. We present a new method for modelling biological invasions using historical spatio-temporal records. This method first discriminates between data points of anthropogenic origin and those originating from natural dispersal, then estimates the natural dispersal kernel. We use the expectation-maximisation algorithm for the first step; we then use Ripley’s K-function as a spatial similarity metric to estimate the dispersal kernel. This is done accounting for habitat suitability and providing estimates of the inference precision. Tests on simulated data show good accuracy and precision for this method, even in the presence of challenging, but realistic, limitations of data in the invasion time series, such as gaps in the survey times and low number of records. We also provide a real case application of our method using the case of *Litoria* frogs in New Zealand. This method is widely applicable across the field of biological invasions, epidemics and climate change induced range shifts and provides a valuable contribution to the management of such issues. Functions to implement this methodology are made available as the R package Biolinv (https://cran.r-project.org/package=Biolinv).

## Introduction

Biological invasions are increasingly common phenomena due to the intensification of transportation of both people and goods [1]. Human aided (anthropogenic) dispersal of invaders occurs at the initial stages of invasions (when species are introduced in new areas) and can persist during the invasion, with people acting as dispersal vector during the colonisation of new areas.

The mechanics of anthropogenic dispersal are clearly different from those of natural dispersal. When modelling biological invasions, however, anthropogenic dispersal is often modelled with the same tools used for natural dispersal: it is often attributed to long distance dispersal (LLD) or modelled as a separate, flat density kernel from the current distribution with a maximum dispersal distance and a frequency of occurrence. These kernel-based approaches result in the anthropogenic dispersal points being clustered around the previously colonised localities. There are a number of situations in which this type of model is suboptimal: (1) when the source of propagules is located in the native distribution range (i.e. multiple introductions), (2) when dispersal is triggered by a request from the receiving end (e.g. fishes introduced for sport-fishing, ornamental plants, or pets–see the Litoria example presented later), or (3) when the speed and range of anthropogenic dispersal is much higher than the natural counterpart (i.e. anthropogenic dispersal could be realistically approximated by instantaneous colonisation of any new locality).

Recent modelling approaches that consider anthropogenic dispersal include Interacting Particle Systems (IPS), like in Engler & Guisan (2009)[2] and Pitt *et al*. (2009)[3], and Bayesian inference, like in Caley *et al*. (2015)[4], Catteral *et al*. (2012)[5], Cook *et al*. (2007)[6]. In both the IPS methods, dispersal is divided into Short Distance Dispersal (SDD) and LDD. The method of Pitt *et al*. has the four neighbouring cells of any occupied cell becoming themselves occupied at the subsequent time-step, leaving it to the grid scale to account for different SDD distances. The method of Engler and Guisan instead, uses a stepwise user-defined dispersal kernel to select the cells that will be colonised at the next time-step. In both methods LDD is modelled by sampling distances from occupied cells and is therefore dependent on the past colonisation. Engler and Guisan sample the LDD distance from a uniform distribution with user-defined maximum at a user-defined frequency. Pitt *et al*. sample from a Poisson distribution, with user-defined mean, the number of LDD events. The dispersal distances are then sampled from a Cauchy distribution. None of the IPS methods provide tools for inference of dispersal parameters. Cook *et al*. and the generalisation on their method presented in Catterall *et al*. adopt a Bayesian approach to infer the invasion dispersal kernel from an on-going invasion time series but do not consider LDD separately and limit their dispersal kernel to the power-law and negative exponential probability functions respectively. Caley *et al*. estimated two dispersal kernels to reflect two different modalities of dispersal: a Weibull distribution kernel to fit natural dispersal and a circular kernel with flat distribution to a maximum distance to fit “invasion” dispersal behaviour; anthropogenic dispersal was explicitly included in their model only as selection of the initial introduction localities.

To avoid anthropogenic points aggregating around already colonised localities, we model them with a uniform distribution. While this is a simplification from the true (unknown) process, detailed modelling will typically be hampered by a relatively small number of anthropogenic dispersal events. We demonstrate the method is robust to the use of the uniform distribution in cases where the true process is non-uniform. Starting from existing spatio-temporal data of an on-going biological invasion, our method initially discriminates natural from anthropogenic points. Based on this categorization, the natural sub-sample is used to infer the natural dispersal kernel. By knowing the proportion of anthropogenic introduction and the natural dispersal kernel, is then possible to make forecasts for the future invasion. This method allows for accounting for habitat suitability and provides a measure of precision in the estimated dispersal kernel.

We tested our method on simulated data to assess whether the performance of all steps of the process are accurate and reliable, as well as resilient to variations in sample size, intensity and pattern of anthropogenic dispersal, and ecological niche width, and survey imperfections–a key feature is that the computations remain tractable even when there are gaps in data collection, and thus uncertainty about the exact time of the dispersal events.

We also used this method on the real case scenario of the introduction of three species of *Litoria* frogs from Australia to New Zealand: *L*. *aurea* (Green and Golden Bell Frog), *L*. *raniformis* (Growling Grass Frog) and *L*. *ewingii* (Brown Tree Frog) have been introduced by acclimatisation societies in the 19th century and currently, *L*. *aurea* occupies the northern part of the North Island while *L*. *raniformis* and *L*. *ewnigii* are found across the two main islands. *Litoria* tadpoles and adults are commonly sold as pets in New Zealand and are frequently traded online with tadpoles being shipped by post across the country. Commonly, unwanted *Litoria* adults are released into freshwater bodies aiding the spread of these species.

## Methods

### Aim of algorithm

Given a dataset with spatial and temporal coordinates (time series) of an exotic species during a biological invasion, this algorithm can be used to answer two main questions: What proportion of points of the invasion time series is of anthropogenic origin? What does the dispersal kernel of the naturally dispersed points look like?

### Method for estimating the anthropogenic component

In order to discriminate between points of anthropogenic and natural origin we used the expectation-maximisation (EM) algorithm [7]. Our main assumptions are (1) that the subset of points of anthropogenic origin can be described by a Poisson point process where the probability of having a new population of anthropogenic origin occurring at any spatial location does not depend on the distribution of the other populations. By contrast, (2) new natural populations are more likely to occur in proximity of other extant populations.

The EM algorithm is based on the nearest neighbour distances *d*_*i*_: for each point in the time series the nearest neighbour distance with other points of the same or previous years is computed.

To avoid overestimating SDD and losing track of human mediated dispersal events, we force at least one nearest neighbour distance per year to be measured against points from past years. The dispersal kernel is then fit to the amended nearest neighbour distances. Take the example of an anthropogenic point from year *i* generating 4 natural points in the period *i*+1 to *i*+2. If no survey is carried out in the area in the period *i* to *i*+2 this set of points would all be labelled *i*+3, and their nearest neighbour distances would all be small, reflecting the erroneous assumption that all of them are of natural origin. Our correction forces one of these points to have a nearest neighbour distance measured against points of year *i*-1 or smaller, thus recovering the otherwise lost, long, anthropogenic dispersal distance.

Anthropogenic points are assumed to be uniformly distributed: to model their distance to the nearest existing point we computed the yearly probability density function for the nearest neighbour distance of a random point, *g*_*y*_*(d)* by measuring its nearest neighbour distance (10,000 replicates) with all the points in the time series till that year. A yearly approach is necessary since the increasing number of points in the time series makes short distances more frequent as more populations accrue.

The nearest neighbour distribution for naturally dispersed points, *f(d*, *σ)* is a single tail Gaussian distribution on positive numbers; an initial guess at the standard deviation is updated as the algorithm progresses. This distribution is a crude approximation to the true nearest neighbour distribution, or indeed the nearest neighbour distribution implied by the dispersal kernel fitted later. Nevertheless, in simulations, it has worked well for categorizing natural vs anthropogenic points. The mixture distribution of nearest neighbour distances, *L*_*y*_*(d)* is then:

$$L_{y}\left(d\right)=\left(1-\pi \right)g_{y}\left(d\right)+\pi f\left(d,\sigma \right),$$

with *π* being the probability of being of natural origin. We use the EM approach [7] to estimate σ, *π*, and a point specific probability of being of natural origin, *W*_*i*_.

The EM algorithm starts by using an initial guess for *π* and σ to estimate the *W*_*i*_, based on the relative likelihood of the natural and anthropogenic distributions for its nearest neighbor distance

$$W_{i}=\frac{\pi f\left(d_{i},\sigma \right)}{\left(1-\pi \right)g\left(d_{i}\right)+\pi f\left(d_{i},\sigma \right)}$$

We then update *π*:

$$\pi =\frac{\sum W_{i}}{n},$$

and σ:

$$\sigma =\sqrt{\frac{\sum \left(W_{i}d_{i}{}^{2}\right)}{\sum W_{i}}}.$$

This process is iterated until the estimates of *π* and σ don’t change more than 0.00001. This process is quite robust to the initial values of *π* and σ.

### Dispersal probability distribution

In order to describe dispersal distance probability distributions we used the *One Dimensional Dispersal Kernel function* [8], which is based on two variables: the shape parameter C and the distance parameter *α*:

f(x)=c2αΓ(1c)exp(−|xα|c),

where Γ indicates the gamma function.

**Fig 1:**
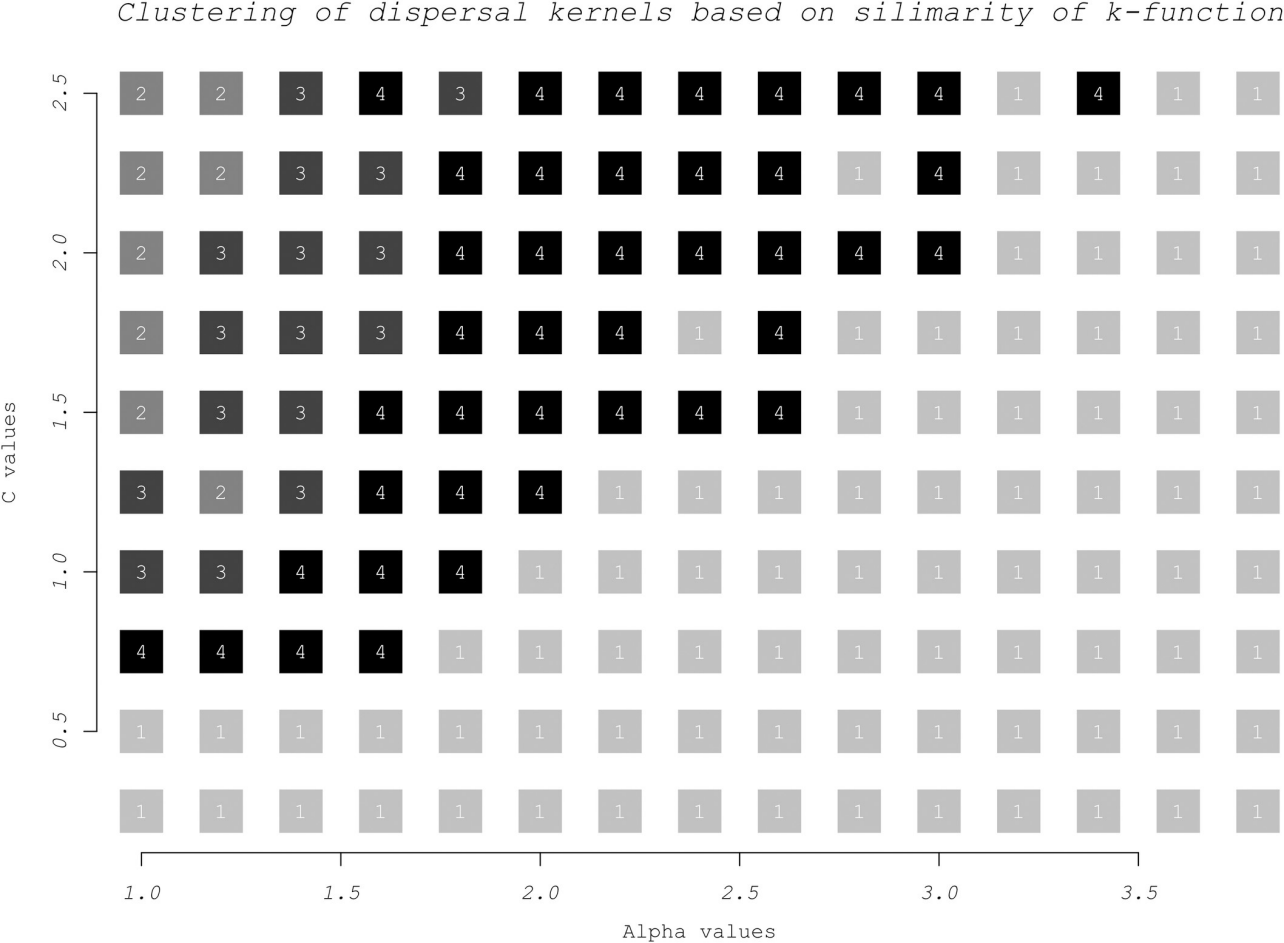
Kernel clusters. How different values of α and C cluster in four groups. x axis clipped at 4; α>4 clusters in group 1.

C accounts for different levels of kurtosis with the negative exponential and the Gaussian distributions being special cases (C = 1 and C = 2 respectively); C<1 makes for a fat tailed kernel. In order to choose which combinations of *α* and C to consider in our algorithm we generated 496 single-generation datasets with all the combinations of *α* (regular sequence from 1 to 7 with 0.2 increment), C (regular sequence from 0.25 to 2.5 with 0.25 increment) and maximum dispersal distance (10). We then performed a K-means cluster analysis on the dissimilarity matrix obtained by computing the sum of squared differences of the Ripley’s K-function [9] of all possible couples of datasets. The number of clusters was chosen by detecting a bend in the plot of the within-group sum of squares (y axis) over the number of clusters (x axis) (S1 Fig, “Choice of No. of clusters”). This analysis highlighted four groups of inherently similar dispersal kernels (Fig 1). Within these groups, different *α* and C combinations cannot be discriminated from one another. Therefore, we are only going to estimate in which cluster *α* and C belong to rather than their actual values. The chosen representatives of each of these clusters are *α* = (10%, 15%, 25% and 50% of the maximum dispersal distance) and C = 2 (Gaussian distribution). Keeping C constant greatly reduces computation time while still being representative of all the four identified clusters.

### Method for estimating the natural dispersal kernel

The EM algorithm is capable of discriminating between natural and anthropogenic populations, however, the nearest neighbours do not necessarily correspond to the source population and for this reason it does not provide an accurate estimate of the dispersal distribution. Another important problem in modelling the dispersal process through nearest neighbour data of an opportunistic dataset is the presence of gap years where no data has been collected (at least in some locations). We bypass these problems by adopting a more phenomenological approach where we summarize the spatial properties of the final product of the invasion process with Ripley’s K-function [9]. This approach discards the some of the temporal information of the invasion time series, but it allows computationally efficient estimation of the dispersal kernel even in the presence of gap years.

A threshold of 0.5 is chosen for classifying points in the invasion time series as either of anthropogenic (*W*_*i*_ < 0.5) or natural (*W*_*i*_ > 0.5) origin. The estimation of the dispersal kernel of the natural subset is then performed by comparing the spatial structure of the observed data with a set of simulated data sets with varying dispersal kernels for the natural component. Many replicates are produced for each candidate kernel. The kernel for the set of replicates which is, on average, most similar to the original data, is then considered as the best approximation of the true dispersal kernel.

As a measure of similarity we use the total sum of squares between Ripley’s K-functions of the original time series and simulated ones. Ripley’s K-function measures spatial aggregation and it reflects changes in the dispersal kernel of a time series. For each point in the data set the K-function counts the number of surrounding points within a series of growing search radiuses. In order to accurately estimate the natural dispersal kernel, points of anthropogenic origin do not have their positions simulated, but are copied as they are from the original to the simulated time series.

Simulated time series are generated sequentially, starting with a subset of the original time series representing the initial distribution (iteration zero) of the species at the year of introduction. For each iteration (year) recorded in the original time series, the points of anthropogenic origin are copied without any modification from the original time series. For that same year, the exact number of points of natural origin recorded in the original dataset is generated from the points which are already present from the previous generations in the virtual time series. Dispersal distances from the source point are sampled from the dispersal kernel being simulated. Points generated/copied in this fashion are then added to the previous generation (initial distribution at the first cycle) and the process is repeated.

As boundary for the point generations we used extracted North and South Island shorelines from the CIA World Data Bank II [10,11] as available through worldHires() command in R package *mapdata*. The same boundary has also been used as *window* in the computation of the K-function as available in package *spatstat* with Ripley's isotropic edge correction [12].

### Habitat suitability

Ecological niche can also influence aggregation in a time series: the narrower the niche the more aggregated the points. In order to account for this phenomenon, it is possible to filter the newly generated natural points with a Habitat Suitability Map (or Model, HSM) where the new natural points are deleted with a probability corresponding to the inverse of the habitat suitability value of the HSM cell they fall into. This is done via an iterative process that ensures the number of simulated points matches the number of observed points in the data set. Each year an excess number of new points are generated and successively filtered as explained above. The number of points generated can be set in the algorithm as a multiple of the actual number of points for that year. After the filtering the remaining points are randomly sampled to generate the exact number needed for that year.

### Jackknife resampling

Jackknife resampling can be used to reduce the effect of outlier points in the time series. This is particularly useful when analysing real world datasets, which come without replicates. Jackknifing can also provide a useful measure of uncertainty around the estimated *α* value for each dataset.

To see whether Jackknifing improves the performance of this algorithm, for each dataset a number of subsamples is taken each containing 85% of the points in the original dataset. Points within the same subsample are sampled without replacement, however, the same point can be present in different subsamples. Each subsample is then processed as a normal dataset. The final *α* estimate is taken to be the average of the estimates of all subsamples derived from the same original dataset.

### Algorithm testing

We tested the algorithm accuracy and precision in estimating the proportion of points of anthropogenic origin and the parameters of the dispersal kernel by running it on simulated datasets. We simulated time series of virtual invasions with populations of anthropogenic origin being sampled randomly within New Zealand mainland and naturally dispersed populations spreading from existing ones with a known dispersal kernel. The process of generating the virtual dataset is sequential: for each cycle (year) a precise number of naturally and anthropogenically dispersed populations is created. We generated two sets of virtual datasets, one for testing the EM algorithm and one to test the estimation of the dispersal kernel parameters.

In order to evaluate the classification obtained from the EM algorithm, we generated datasets with varying *α* values (4.5; 15), C values (0.3; 1; 2), proportion of anthropogenic points (0%; 10%; 30%). Each replicate of the virtual dataset is then duplicated and, for one copy, the year names are aggregated in groups of 5 (e.g. 1, 2, 3, …20 becomes 5, 5, 5, 5, 5, 10, 10, 10, 10, 10, 10, …, 20, 20, 20, 20, 20). This was done to simulate the periodic sampling effort of real world data collections. To test for the EM algorithm robustness to deviations from the assumption of spatial uniformity in the anthropogenic subset, we replicated all the virtual datasets described above, making the intensity of the process generating anthropogenic points proportional to a measure of road density. All virtual datasets were run through the EM algorithm and all point were classified as either natural or anthropogenic. We then compared the EM-classified values against the original values to determine the proportion of points correctly classified. When the true proportion of anthropogenic points was non-zero, we also computed the ratio between the estimated and original proportions to assess over/under estimation.

To test the dispersal kernel estimation, we generated a set of datasets that varied in key attributes. For each parameter setting, ten replicates were generated. All combinations of the following were tried: *α* (4.5 or 15), total number of points (100, 400, 1000, 2000), percentage of points with anthropogenic origin (0, 0.05, 0.1, 0.3) and amount of aggregation (without aggregation, or aggregation into 5 year bins). In each case the number of generations (years) was (80), the maximum natural dispersal distance was 30Km, and C = 2. Every virtual dataset generated was then run through the algorithm and the quality of the *α* estimate was measured as the difference between the estimated and real value. *α* values used in the comparison datasets were 2; 3; 4.5; 7.5; 11; 15; 20; and 25; for each value 90 replicates were generated.

To test the effect of the HSM filtering we generated a virtual dataset where, for every cycle of the point generation algorithm, new points (anthropogenic and natural) are produced in higher quantity than needed and subsequently filtered with the HSM for *Litoria raniformis*. This virtual dataset is then processed with and without accounting for habitat suitability and the results are compared.

To test in what measure Jackknife resampling improves the performance of the algorithm we took two sets of ten replicate datasets that produced relatively inaccurate results and performed 10 85% Jackknife resampling on each replicate. All the Jackknife datasets have then been processed as described in the “Jackknife resampling” section, and their *α* estimates compared with the results of the procedure without Jackknifing.

### Applied example

To check whether the expansion of *Litoria* species in New Zealand has already reached its maximum, a MaxEnt (MaxEnt version 3.3.3k [13]) Habitat Suitability Model (HSM) was built for each species based on the sighting locations retrieved from New Zealand’s Department of Conservation (DoC) Amphibians and Reptiles Distribution Scheme (ARDS). The database records have been selected in order to remove duplicate records (same combinations of species, latitude and longitude) and data with an inaccuracy of more than one km. Accuracy in ARDS is classified into 7 classes; imprecision less than 5Km (model cell size) would be acceptable, but data with more than one Km of error are classified in the next class of 10Km inaccuracy and were therefore discarded. After this process, there were 129 records for *L*. *aurea*, 254 for *L*. *raniformis*, and 323 for *L*. *ewingii*.

Pearson’s statistic was used to test the spatial correlation of a total of 31 environmental variables that could potentially be included in the model (elevation; percentage of artificial forests; all 19 Worldclim biological variables; percentage of cropland; grassland (percentage of grassland; LUCAS 2^nd^ edition); percentage of natural forest; density of roads; slope; solar radiation; percentage of urban area; percentage water cover; percentage of wooded grassland (see Table 1 in S1 File for variables’ source). Only a subset of 14 variables with low pairwise correlations (-0.7<p<0.7) were kept for the analysis (Table 1 in S1 File). All environmental variables maps have projected in an equal area coordinate system (New Zealand Transverse Mercatore 2000 (NZTM2000). All maps have been given the same spatial extent, pixel size and corner coordinates and were saved as ASCII files (No. of columns: 1000; No. of rows:1371; No. of cells: 1371000; Extent: 1089971, 2089971, 4823127, 6194127 (xmin, xmax, ymin, ymax); Cellsize: 1000 x 1000; Length measure of unit: meter).

To correct for the clustering of sighting locations due to systematic sampling errors (i.e. sighting locations are more abundant close to cities or research sites), a background dataset of 10’000 random points stratified on a kernel density estimate of all the sighting locations of the three species together was used. Cross-validation of MaxEnt models has been performed over 10 random sub-samplings of the original dataset. A Multivariate Similarity Surface (MESS) has been computed to highlight the areas of novel habitat type compared to the current distribution of the species.

### EM algorithm and kernel estimation

Jackknife resampling was done on the cleaned ARDS time series for the three species of *Litoria* frogs in New Zealand. Ten Jackknifed datasets, each of which contain 85% of the data points, were obtained and processed in parallel.

The EM algorithm was run on the Jackknifed datasets and on the original full dataset. The 10’000 random points used for the estimation of the yearly probability density function for the nearest neighbour distances were sampled from the whole of New Zealand for *L*. *raniformis* and *L*. *ewingii*, while for *L*. *aurea* the points where sampled exclusively within the North Island as there are no sighting locations for this species in the South Island. Points were considered of anthropogenic origin when their probability of being so (1 –prob. of being natural) was bigger than 0.5.

Once the origin of the population was classified as either natural or anthropogenic the dataset was run through the dispersal kernel estimation algorithm. Spatial filtering was used to account for habitat suitability. The number of points produced each year was set to be 30 times the actual number needed. MaxEnt models described above were used as the HSM. The number of replicates for the comparison datasets was set to 90 and their initial *α* values were 2, 3, 4.5, 7.5, 11, 15, 20 and 25. *α* values were updated to higher numbers ((2, 5, 10, 20, 30, 40, 50) and (20, 30, 40, 50,60,80,100)) for those datasets who required them in order to identify a minimum dissimilarity in the range of tested *α*s (“U” shaped lines in the similarity plots of the results section). The first ten rows of each Jackknifed dataset (the oldest ten points available) were used as the initial dataset on which to build the comparison datasets. The search radius for Ripley’s K-function was set from zero to 30 km (and, if needed, 100 and 200km) at one km steps.

## Results

### Algorithm testing

**Fig 2:**
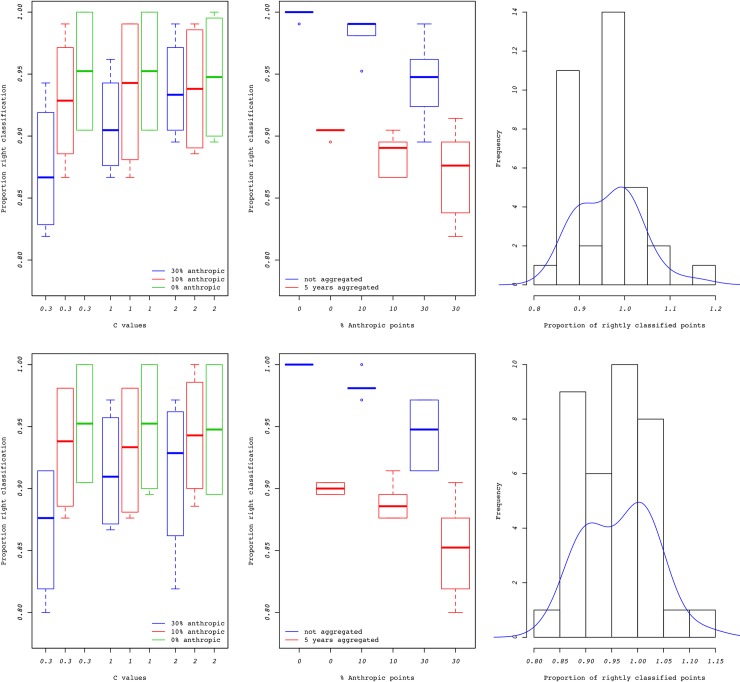
EM classification accuracy. Proportions of rightly classified points and estimated vs. real proportion of anthropogenic points from the EM algorithm. First row: spatially uniform anthropogenic points. Second row: anthropogenic points aggregated around areas of high road density.

Assessment of the EM classification showed an overall high proportion of correct classification although yearly aggregation, C value, and the proportion of anthropogenic points had clear effects on the proportion correct classification (Fig 2). A combination of high percentage of anthropogenic points and leptokurtosis causes the lowest success rates in the EM classification. In these conditions, the algorithm confuses some anthropogenic points with natural LDD events. Similarly, a high percentage of anthropogenic points confuses the EM classification in the presence of gap years. The impact of non-homogeneous intensity for the anthropogenic points was small. Fig 2 shows that, when the true anthropogenic proportion is non-zero, errors in classification typically result in underestimation of the proportion of anthropogenic points.

**Fig 3:**
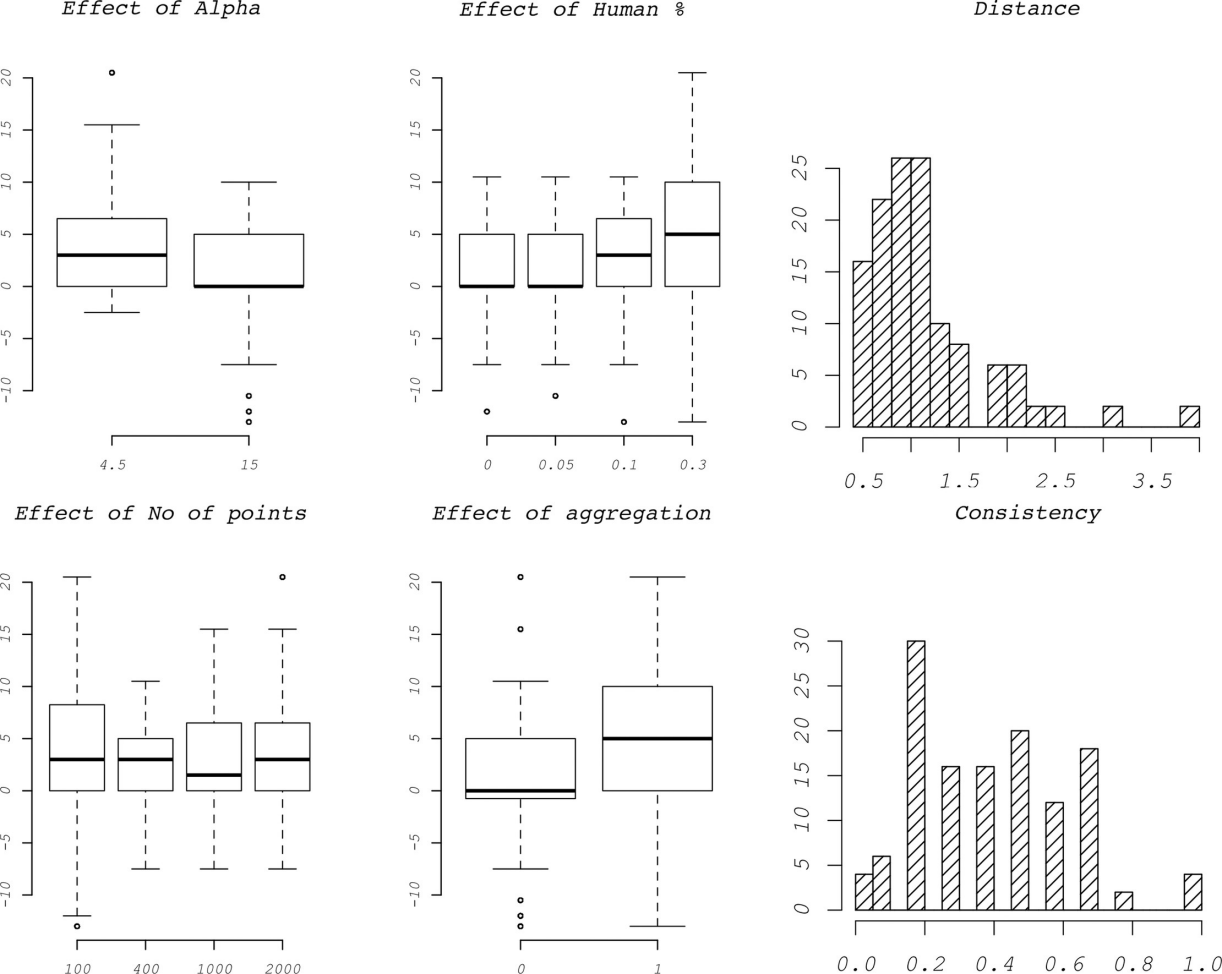
Performance of α estimation. **Boxplots**: Summary of the effect of different variables used to build the virtual datasets on the performance of the algorithm. “**Distance**”: Histogram showing the average of how many α values away from the mean each replicate is. 1 means that, if the real is 15, that replicate is one step away along the sequence of tested values (2; 3; 4.5; 7.5; 11; 15; 20; 25) and therefore it could be either 11 or 20. “**Consistency**”: Histogram of the proportion of replicates that result in the same α estimate as the mean over replicates.

**Fig 4:**
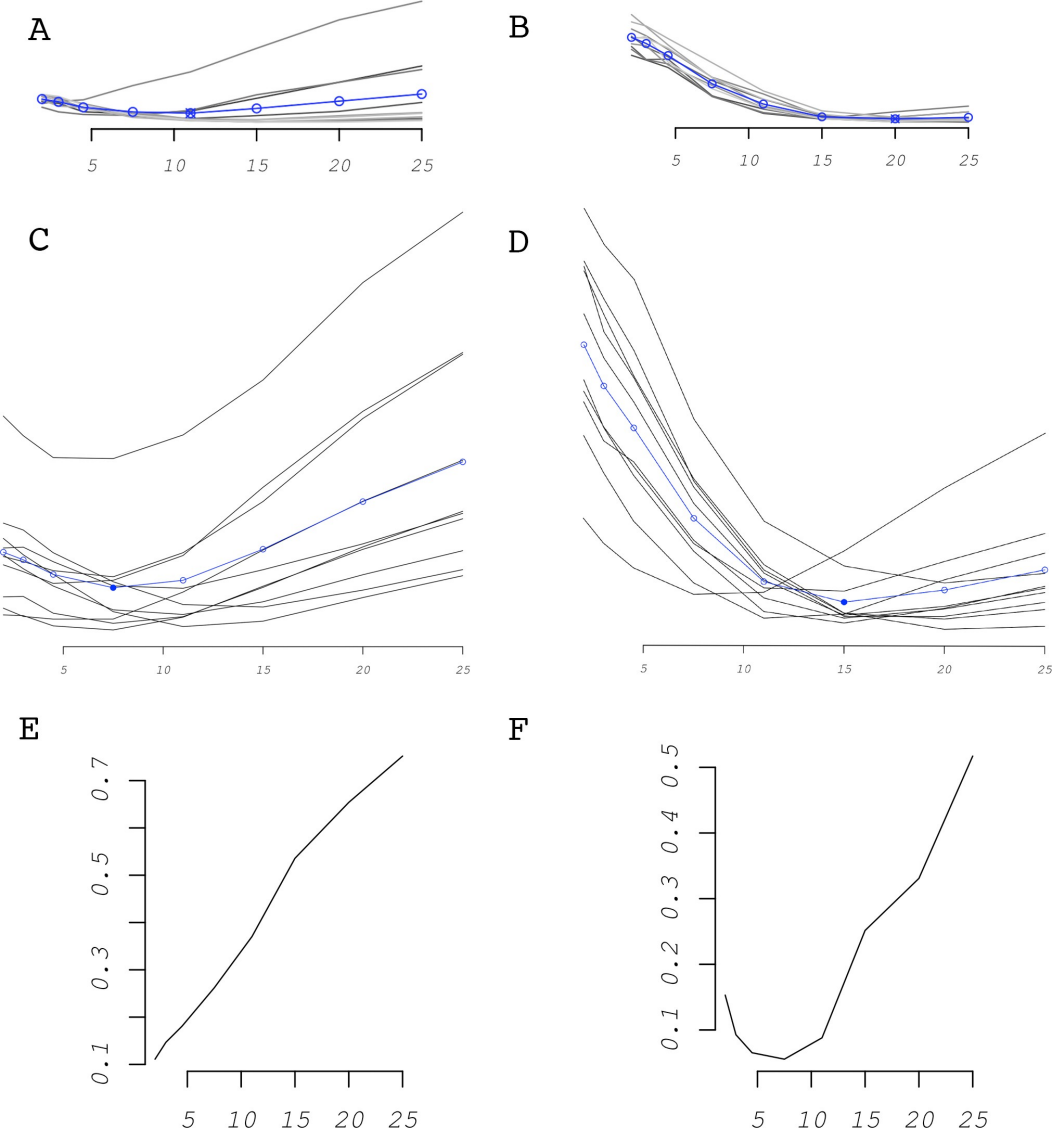
Performance of Jackknife re-sampling and HSM filtering. “**A-D**”: Effect of Jackknife re-sampling on two data sets (ten replicates) that produced poor results (“**A**” & “**C**”: α = 4.5, % anthropogenic = 10, n = 100, no aggregation. “**C**” & “**D**”: α = 15, % anthropogenic = 30, n = 400, with aggregation). “**A**” & “**B**”: without Jackknife; “**C**” & “**D**”: with Jackknife. “**E-F”:** Comparison of one virtual data set (α = 4.5; hd = 0.1; n = 400; gen = 80) simulated with L. raniformis’s HSM and analysed with (F) and without (E) HSM filtering.

Of the 32 variations on the virtual datasets (average of the replicates, S2 Fig) 18 *α* estimates fell within the right cluster of *α* and C combinations, eight are overestimated by one unit and six were overestimated by two units. Within the 16 virtual datasets with *α* = 15, only two *α* estimates were off by one step, while all the others were exact. Virtual datasets with 400 points produced better estimates of *α* than those with 100. In Fig 3 (“*Effect of Alpha*”), outliers for simulated datasets with *α* values of 4.5 are only above–and only below for datasets with *α* of 15. This may be due to the sequence of tested *α* values (2; 3; 4.5; 7.5; 11; 15; 20; 25) being relatively longer below for 15 and above for 4.5. Jackknife resampling improved the overall estimation of the dispersal kernel and reduced the variability between replicated datasets (Fig 4 “*A-D*”). Improvement was from two units overestimation to one unit overestimation for the first dataset and from one unit overestimation to exact estimate for the second dataset.

Increasing the proportion of anthropogenic populations increases the variability of the estimation but does not increase bias. A similar effect is generated by the decreasing number of points in the datasets, where the main change is the reduction in precision, but not in accuracy, of the *α* estimate with datasets with only 100 points. Introducing in the dataset gaps years (aggregation), also causes a small increase of the estimation’s variability (Fig 3, “*Effect of aggregation*”).

The HSM filtering dramatically improved the accuracy of the algorithm. Spatial clustering, in virtual datasets where spreading is constrained by habitat suitability, is overestimated if the ecological niche of the species is not considered (Fig 4 “*E*” and “*F*”).

### Applied example

**Fig 5:**
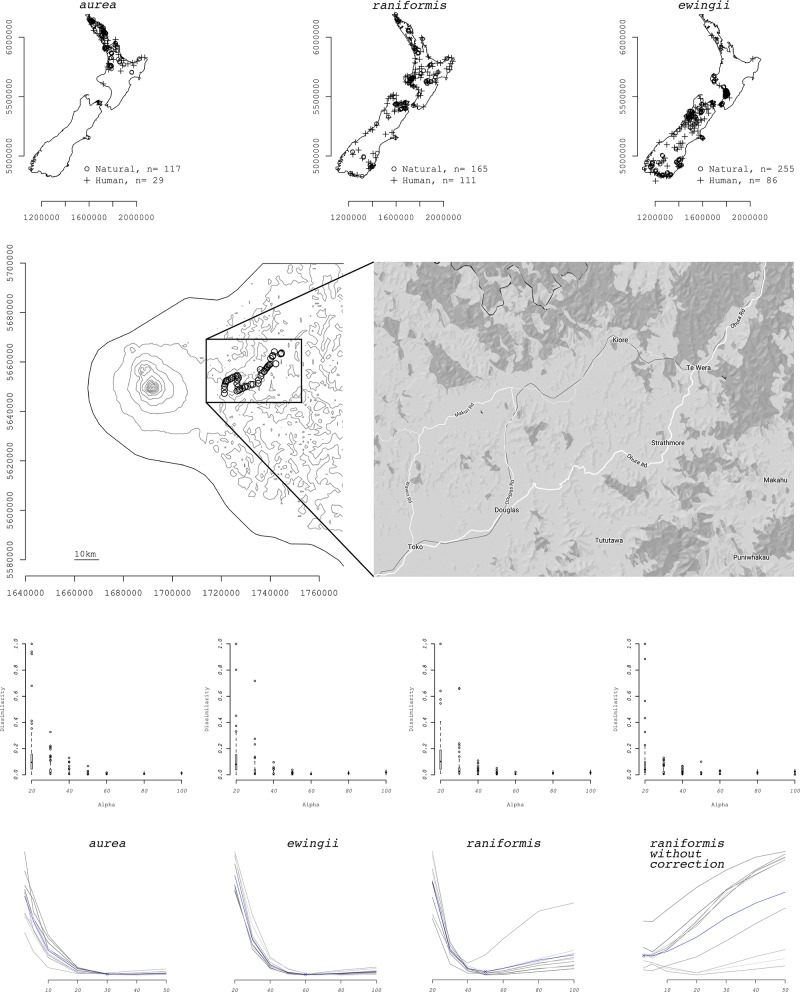
Applied example, Litoria spp. in New Zealand. **First row**: Maps of sighting locations for Litoria frogs in New Zealand after EM classification: populations of human origin as crosses and of natural origin as circles. **Second row**: Artefact in the dataset of L. raniformis that was removed. A line of very dense sighting locations along a road in Taranaki from 2001. On the right an enlargement of the road followed during the survey. **Third row**: Dissimilarity values between comparison and observed datasets for the Litoria raniformis time series. Each boxplot represents similarity values of all simulated datasets with the same α value (sample of four out of ten Jackknifed datasets; see S3 Fig for all Jackknifed datasets). Each plot is a Jackknifed dataset. **Fourth row**: Average similarity values between comparison and observed datasets for the Litoria spp. time series. Each line represents averaged similarity values of all the simulated datasets for each α value. Each line represents a Jackknifed dataset. HSM filtering was performed. The blue line is the average of the grey lines.

Results of MaxEnt modelling are available in S1 File. The EM algorithm classified 19.86% (29 out of 146) of *L*. *aurea* populations, 40.22% (111 out of 276) of *L*. *raniformis* populations and 25.22% (86 out of 341) of *L*. *ewingii* populations as of anthropogenic origin (Fig 5, first row).

The estimated dispersal kernel for *L*. *aurea* is *α* = 30 km; *L*. *ewingii* is estimated to disperse with *α* = 60 km. Both these species present a “U” shaped similarity plot (Fig 5, fourth row “*aurea*” and “*ewingii*”). The dispersal kernel for *L*. *raniformis* is a positive linear function with estimated value of *α*: 2 km (Fig 5, fourth row “*raniformis without correction*”).

The results for *L*. *raniformis* however show more variation between Jackknifed datasets compared to the other two species, with some datasets, having a very different behaviour (almost opposite) to the one of the majority of the other Jackknifed datasets. Such a different estimation of the dispersal kernel for such similar species (especially *L*. *aurea*) seems to point at an artefact in the dataset. For this reason, 49 points classified as natural origin in the dataset for *L*. *raniformis* were deleted. These points are very close to one another and follow a road in Taranaki (Makuri and Wawiri road, Fig 5; they all date 2001). These points are obviously part of a specific survey where all animals sighted along the road were passed to the ARDS and included in the general dataset. After the deletion, the new dataset was run as the previous and results changed radically with *α* now being estimated as 50 km and the similarity plot being “U” shaped, similarly to the other species (Fig 5); the proportion of anthropogenic points is now 34.80% (instead of 40.22%) as 32 anthropogenic and 17 natural points were deleted (misclassification of few of these points occurred as a consequence of being an artefact). Also, the new dataset has all the Jackknifed replicates behaving similarly (Fig 5, fourth row “*raniformis*” and “*raniformis without correction*”). It is not necessary to remove this survey artefact prior to the building of the HSM because all of the points fall in two single raster cells and are therefore automatically reduced to two single points by the MaxEnt algorithm.

## Discussion

Our method’s accuracy for both the classification of anthropogenic vs. natural populations and the estimation of the dispersal kernel is reliable and can be informative for datasets with as little as 100 records, even when there are gaps in the survey years and when the assumption of spatial uniformity of the anthropogenic points is violated.

Single *α* estimates have a certain variation around their mean, but most values are just one step away along the sequence of tested *α* values (Fig 3).

We suggest using the HSM implementation whenever possible as our virtual dataset test shows it drastically improves the accuracy of the algorithm.

The anthropogenic contribution to the spread of *Litoria spp*. in New Zealand was inferred to be very high, with an average of ~27%, and peaking at ~35% in *L*. *raniformis*, of the recorded populations having originated through anthropogenic dispersal. Although a minority of individual records are of anthropogenic origin, most of the large scale spread is inferred to be anthropogenic in nature. This is highly plausible based on the pet trade in these frogs.

Jackknifing is also a very useful implementation as it 1) gives more accurate results, especially for datasets that are small or potentially flawed; 2) provides information on the variability of the estimated dispersal kernel and 3) helps detecting the presence of inhomogeneous areas in the source dataset. In the case of *Litoria spp*. Jackknife analysis helped detecting a survey artefact that otherwise would have caused a major bias in the estimation of the dispersal kernel. This was highlighted by Jackknifed datasets behaving very differently from the others when a good portion of the survey artefact was randomly removed by the Jackknife process.

Although we used the generalised assumption of spatial uniformity in the anthropogenic subset in the EM algorithm, our methodology has been proven robust to departures from this assumption. Theoretically, the more aggregated the anthropogenic points are, the more difficult would be for the EM algorithm to detect them as their probability distribution may start resembling that of natural dispersal. However, the bigger scale at which anthropogenic dispersal operates reduces the chances of such an aggregation. A notable exception is when the destination sites are aggregated (e.g. the density of commercial landscaping suppliers for the case of the Cane toad in Australia [14], or specific maritime trades for Mediterranean alien species [15]), this, however, also make anthropogenic points easy to identify without any elaborate modelling.

We modelled anthropogenic dispersal as a spatially homogeneous process to include those scenarios that behave as multiple, ongoing introductions of a species (a dispersal kernel approach has difficulty capturing this behaviour). However, nothing prevents anthropogenic dispersal types which depend on past colonisation points to be modelled as LDD in the natural dispersal kernel, thus making our method suitable for multiple types of anthropogenic dispersal.

Once the dispersal kernel and the proportion of new releases is known, the same method used to produce simulated datasets can be used to project any invasion to into the future. In the case of *Litoria spp*. this is not particularly useful as the boundaries of the potential distribution have been practically reached already; projecting the invasion would generate new points whose density would merely reflect the HSM. Projecting an invasion into the future however, is of great importance especially at the early stages of biological invasions. When doing this, it is fundamental to remember that the process our method is modelling is not necessarily the actual spreading of the study species, but rather the spatial point process in the sighting locations database. Only if the survey is thorough enough to approximate the real invasion (equally surveying across different environments, locations, and seasons, with good spatial and temporal coverage), can we expect to be modelling the actual invasion. For this reason, especially when the sighting locations database is not particularly thorough, the estimated dispersal kernel is to be handled carefully outside the framework of phenomenological/statistical invasion modelling (e.g. in mechanistic dispersal models). Additionally, when the anthropogenic subset is thought to be aggregated around some underlying spatial variable (e.g. road density), during the forecasting phase it is appropriate to sample future anthropogenic points from a sample stratified around such variable rather than a uniform one.

Future developments of this method would include accounting for climate change both in the dispersal kernel estimation and in future projections. This would also render the methodology more appropriate for the estimation of climate-change-induced range shifts, where cells de-colonisation needs to be considered. It would also be possible to implement a release probability map, for use in the identification of anthropogenic populations and for future projections. Classification of the anthropogenic points might also be improved by feeding into the EM algorithm an estimate of the nearest neighbour distribution derived from the inferred natural dispersal kernel rather than imposing a normal shape. In addition to biological invasions, this method could be investigated for use modelling epidemics, as long as spatio-temporal data on the on-going process is available.

Functions to implement the methodology presented in this paper are made available as the R package Biolinv (https://cran.r-project.org/package=Biolinv, see S2 File for a tutorial with the analysis of a sample dataset).

## Supporting information

S1 File MaxEnt models’ details. Detailed information about MaxEnt models’ parameters and results, with images.
(DOCX)

S2 File Biolinv R package tutorial. Biolinv package tutorial with the analysis of a sample dataset.
(DOCX)

S1 Fig Clustering of the one-dimensional dispersal kernel. “Choce of No. of clusters”: Plot of the within-groups sum of squares against the number of groups for choosing the number of groups for the cluster analysis. “Representatives of the four clusters”: Few dispersal kernels grouped in their respective cluster.
(PNG)

S2 Fig Dissimilarity curves. Dissimilarity curves of the α estimation of all 320 virtual datasets.
(PNG)

S3 Fig Dissimilarity values for *L. raniformis*. Dissimilarity values between comparison and observed datasets for the *Litoria raniformis* time series. Each boxplot represents similarity values of all simulated datasets with the same α value. Each plot is a Jackknifed dataset. HSM filtering was performed.
(PNG)
