# Supplementary material for: A new method for modelling biological invasions from early spread data accounting for anthropogenic dispersal
Source: PLoS One. 2018 Nov 27;13(11):e0205591. doi: 10.1371/journal.pone.0205591 (PMC6258513; doi:10.1371/journal.pone.0205591)
Supplement: S1 File — Detailed information about MaxEnt models’ parameters and results, with images. (DOCX) [file pone.0205591.s001.docx]

# S1 appendix, MaxEnt

### MaxEnt parameters

Testing on the *Litoria spp.* MaxEnt models, was done by 10-fold cross-validation. 10% of the sighting locations were kept aside for testing. Response curves were created for all the variables both as a single-variable MaxEnt model and with all variables together. Permutation importance of environmental variables was also computed: for each variable in turn, the values of that variable on training and background data are randomly permuted, the model is re-evaluated on the permuted data and the resulting drop in training AUC (Area Under the receiver operating characteristic Curve, the probability that any sighting location cell is given a higher habitat suitability value then any background cell) is plotted. After a trial run, “Autofeatures”, “Threshold” and “Hinge” feature types were disabled to avoid overfitting. Jackknife resampling of the environmental variables was used to test the variables importance.

The used output format was “Raw” and the maximum number of iteration was fixed at 10’000 but never exceeded 1’500 for *L. raniformis*, 1640 for *L. ewingii* and 2040 for *L. aurea*. Model performance was tested by computing the AUC for classification of location cells as sighting locations.

### MaxEnt results

Habitat suitability models’ performance, as indicated by the AUC values, is good: *L. aurea* 0.835 (σ 0.026); *L. raniformis* 0.773 (σ 0.027); *L. ewingii* 0.790 (σ 0.034). Results of the MESS analysis show that the only case for which there is a significative area of novel habitat is *L. aurea* (Appendix S1: Fig S1). As shown by the MaxEnt models (Appendix S1: Fig S2), this area does not overlap with any area of high habitat suitability.

Summarizing the information in Appendix S1: Fig S3-S5 (Jackknife test of the gain contribution of single variables, response curves and variables permutation importance respectively), the distribution of *L. aurea* is mainly driven by the variable “solar”, selecting therefore areas of high solar radiation. Precipitation seasonality (“bio15”) and isothermality (“bio03”) are other important factors, with this species selecting for stable environments in terms of temperature, but with a moderate seasonality in terms of rainfall. This probably reflects the need for water in the breeding season. *L. aurea* is limited in its altitude gradient to about 1000 m.a.s.l.. The most significative variables predicting the presence of *L. raniformis* are “natforest” and “roadkern”, with the species avoiding natural forest and never reaching areas which are far from roads. Also, for this species, habitat suitability decreases with altitude, with a maximum of 2000 m.a.s.l. as the highest tolerable altitude. *L. raniformis* also seems to select flat terrains (“slope”). *L. ewingii* appears to be the more generalist of the tree species, not showing big differences among the importance given to different environmental variables. Its distribution is linked to cities of the least densely populated areas of the country (high “urban” values and low “roadkern” values). Also, this species selects low values of solar radiation (“solar”) and precipitation (“bio15”). Presence of fresh water (“water”) is selected here more than in the other two species.

Models of all the introduced *Litoria* species show areas of un-colonised, suitable habitat mainly within the current distribution range. *L. ewingii* is the species with the biggest area of possible range expansion, while *L. raniformis* presents an amost realised distribution range. Of course, this does not mean that population densities within the range cannot grow.

### Tables and figures

| **Abbreviation** | **Meaning** | **Source** | **Type** |
| --- | --- | --- | --- |
| Altitude | Altitude [m.a.s.l.] | Worldclim (Hijmans *et al.*2005) | Continous |
| Artforest | % of artificial forest | LUCAS 2^nd^ edition (Newsome *et al.* 2013) | Continous |
| Bio02 | Mean temperature diurnal range | Worldclim | Continous |
| Bio03 | Isothermality | Worldclim | Continous |
| Bio12 | Annual precipitation | Worldclim | Continous |
| Bio15 | Precitpitation seasonality | Worldclim | Continous |
| Cropland | % of cropland | LUCAS 2^nd^ edition | Continous |
| Natforest | % of natural forest | LUCAS 2^nd^ edition | Continous |
| Roadkern | Kernel estimate of road density[0,1] | NZ Roads: Road Section Geometry (LINZ) | Continous |
| Slope | Land slope [%] | Worldclim | Continous |
| Solar | Solar radiation [MJ m^-2^ day^-1^] | LENZ (Leathwick John *et al.* 2002) | Continous |
| Urban | % of urban area | LUCAS 2^nd^ edition | Continous |
| Water | Presence/absence of water | LUCAS 2^nd^ edition | Categorical |
| Wdgrassland | % of wooded grassland | LUCAS 2^nd^ edition | Continous |

Table 1: Environmental variables used in the MaxEnt models of the Litoria spp.


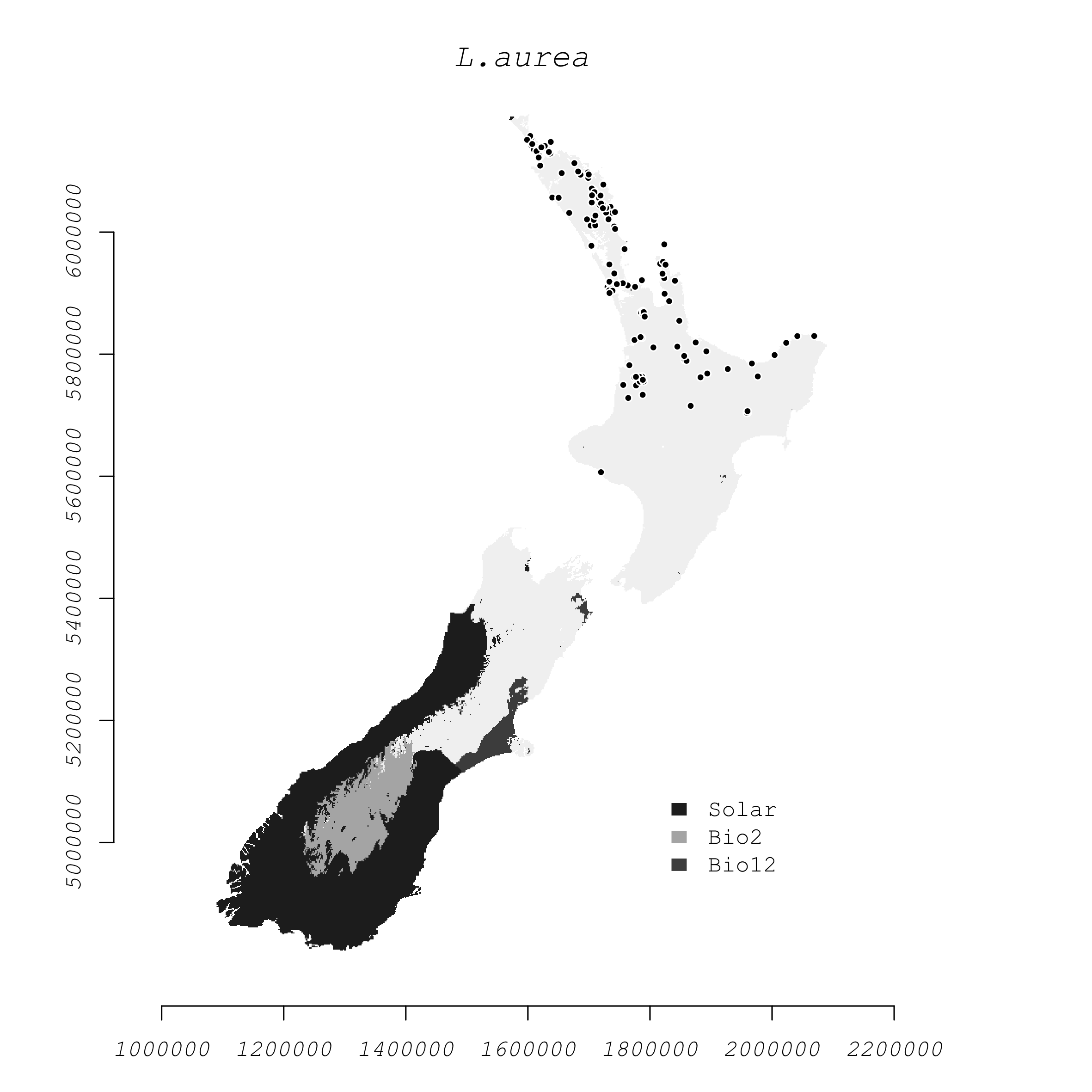


Fig 1: Results of the MESS analysis for L. aurea. Shaded areas have at least one environmental variable outside the training range of the model. Different shades indicate the variable with the most dissimilar values compared to the training range.


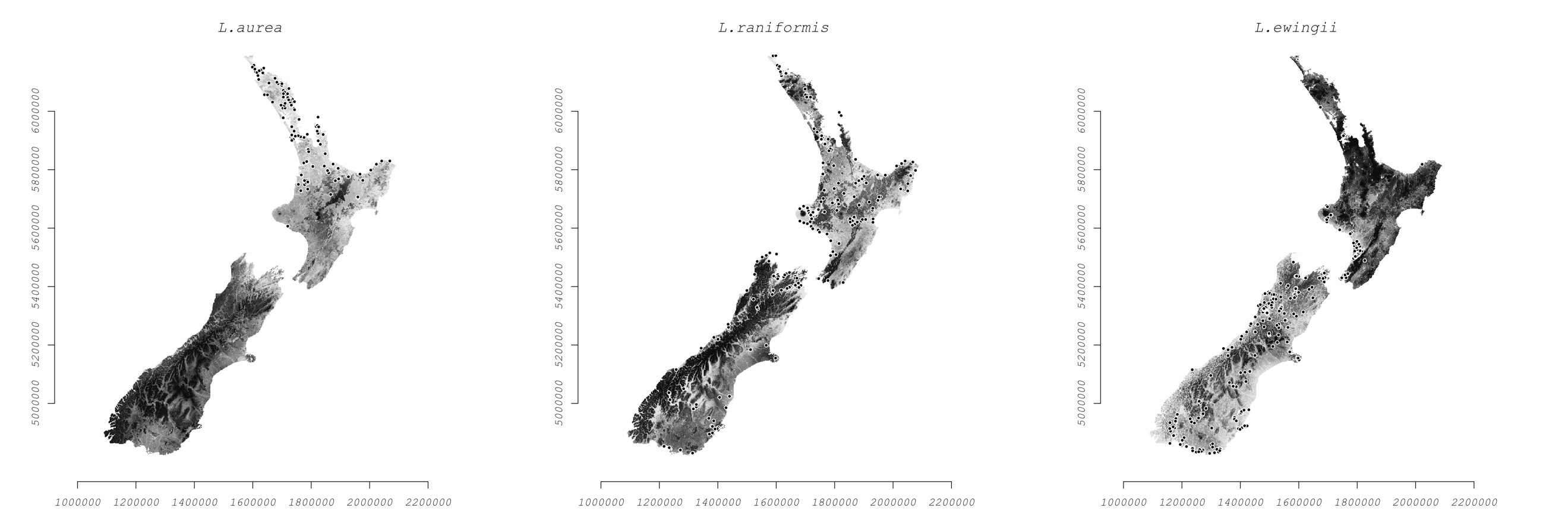


Fig 2: MaxEnt models of Litoria frogs plotted on a quantile scale. Higher values are represented by lighter shades.


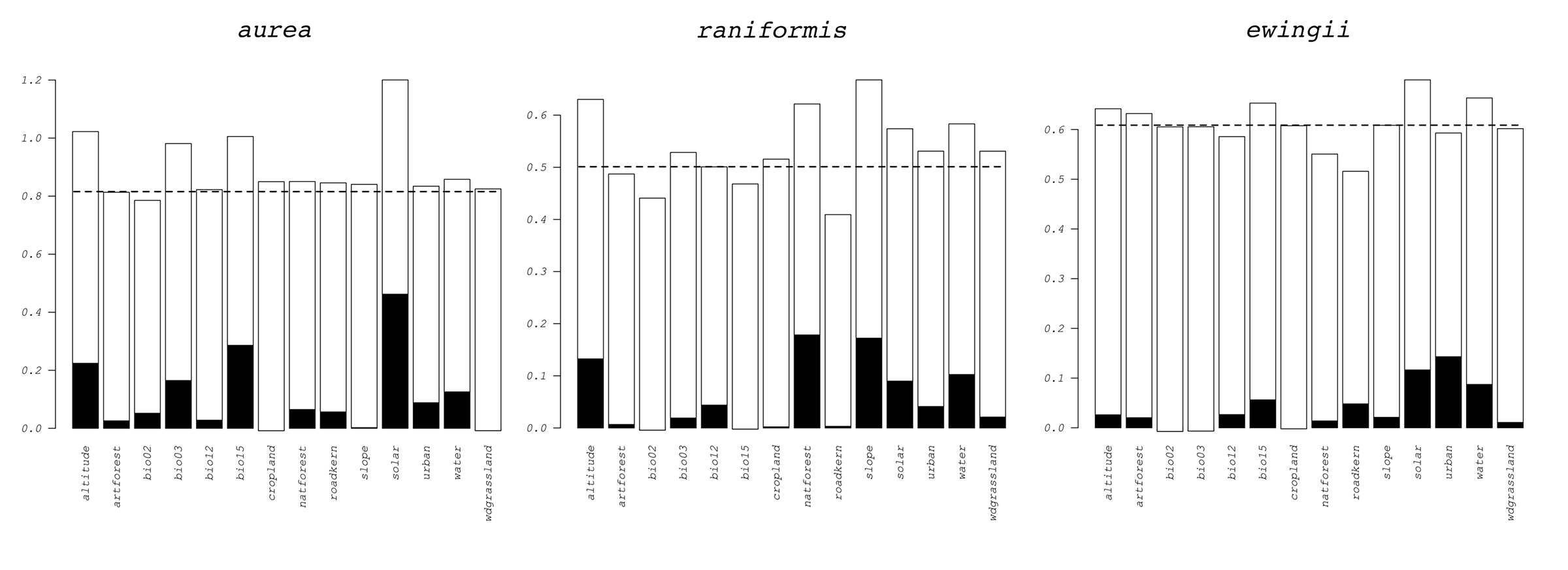


Fig 3: Test gain of the model (on the 10% test data subsample) for each variable. White: on the model without the variable. Black: on a model with only that variable. Dashed line: Gain of model with all variables.


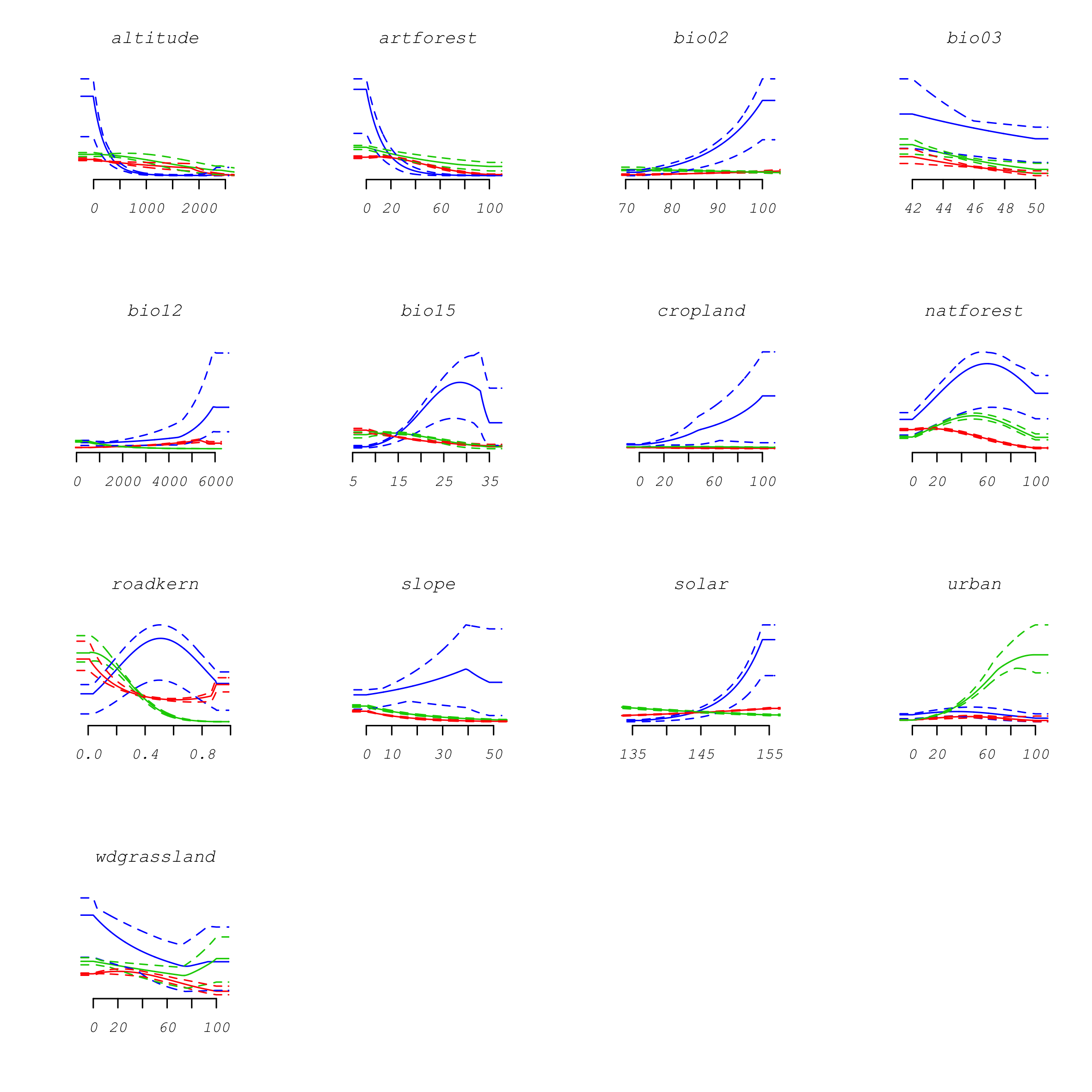


Fig 4: Response curves for all environmental variables computed on MaxEnt models with a single variable per model. L. aurea in blue; L. raniformis in red: L. ewingii in green.


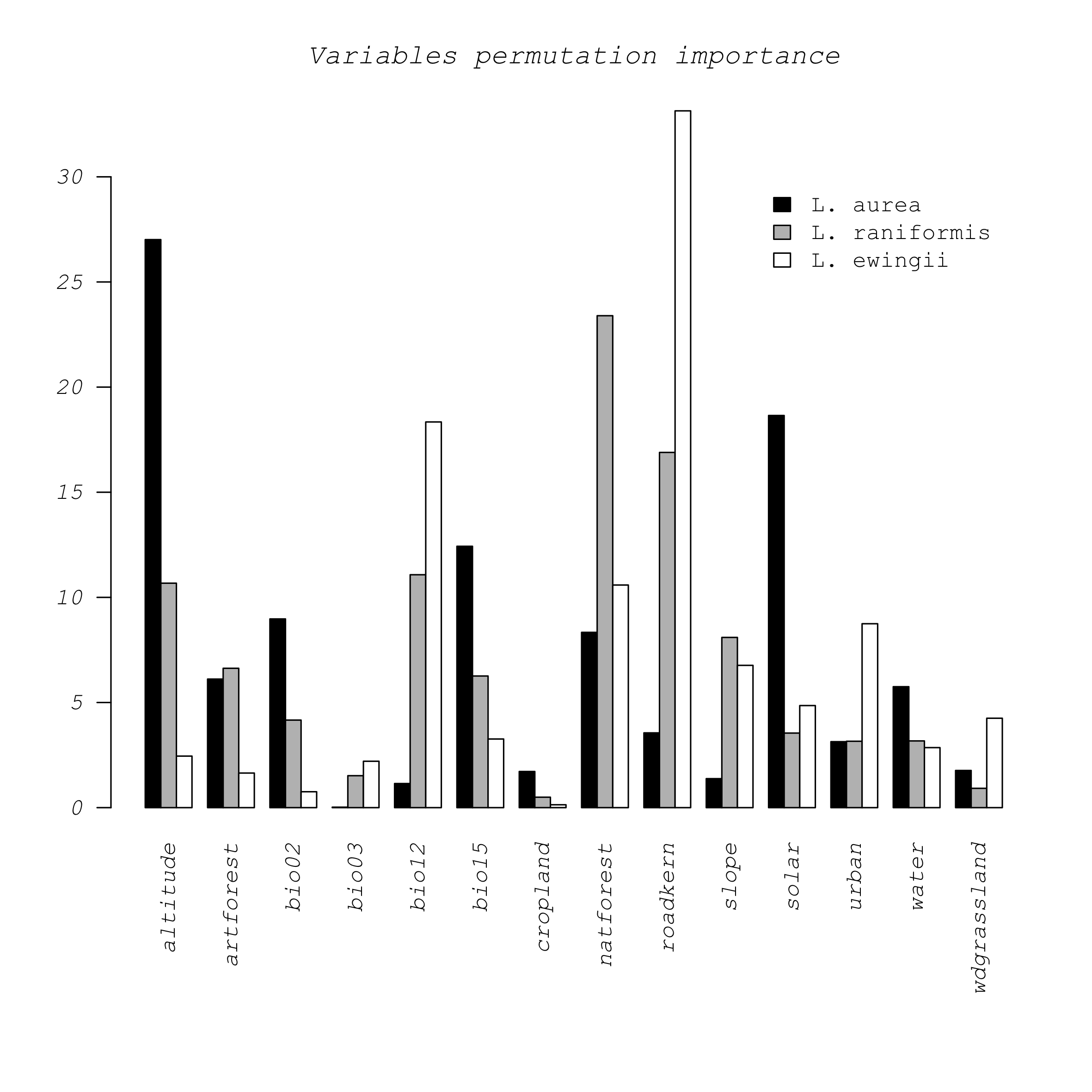


Fig 5: Permutation importance of single environmental variables for the three species of Litoria.
