# Supplementary material for: A new method for modelling biological invasions from early spread data accounting for anthropogenic dispersal
Source: PLoS One. 2018 Nov 27;13(11):e0205591. doi: 10.1371/journal.pone.0205591 (PMC6258513; doi:10.1371/journal.pone.0205591)
Supplement: S2 File — Biolinv package tutorial with the analysis of a sample dataset. (DOCX) [file pone.0205591.s002.docx]

# S3 appendix, Biolinv R package tutorial

### Introduction

The biolinv R package provides tools for modelling and forecasting biological invasions with special consideration to anthropochory, the transportation of propagules by man. Anthropic dispersal is at the core of biological invasions. However, available software that considers anthropochory often models it as long distance dispersal. This implies that the new release sites for anthropic dispersal depend on the location of existent colonised sites, which is not necessarily true in common scenarios (e.g. when the source of propagules is the native distribution range or when anthropic dispersal is much faster than natural dispersal).

Starting from an ongoing invasion time series, Biolinv provides tools for classifying sighting locations that result from either natural or anthropic dispersal. It also estimates the anthropic contribution to the invasion, the invasion’s natural dispersal kernel, and makes future forecasts on the invasion process. This is done while accounting for habitat suitability in the form of a Habitat Suitability Model (HSM, in raster format) and by providing a measure of precision in the estimates.

### Core functions

The package is based on three main functions – namely EM, simulacro and modsel. These are used to run the EM algorithm, generating a virtual dataset and measuring spatial similarity respectively. The package also has several auxiliary functions that are primarily called by the main functions, but they can also be run on their own for specific tasks. Functions for the graphical representation of results are also available.

###### Function EM

The EM function is used to estimate which populations in a biological invasion’s time series are of natural origin and which ones originated by anthropic dispersal. It uses nearest neighbour distances from the invasion time series as computed internally by the function.

The EM algorithm at the core of this function iterates between updating the distribution of nearest neighbour distances for natural points (assumed to be from a half normal (0, sigma), and updating the probability that each point is the result of natural dispersal (Pnat). Starting values for Pnat and sigma must be supplied, but the algorithm is robust to the exact values. The anthropic subset is assumed to come from a uniform distribution; the resulting distribution of nearest neighbour distances (with neighbours assumed to be extant points) is computed by simulation, using the observed points as the extant points and the specified spatial boundary as described in chapter 2b. The output of this process is a vector of probabilities of being of natural origin (Pnat) for each data point. A threshold can then be used to classify points as natural or anthropic.

###### Function simulacro

The simulacro function is used to simulate an invasion time series based on spatial boundaries, a proportion of natural vs. anthropic points, a natural dispersal kernel and a HSM. It can do this wither by simulating “from scratch”, or by copying the anthropogenic release sites from observed data. Time series are generated by an iterative process for which every cycle represents a time step (e.g. one year). For each iteration (time step), a number of points of anthropic and natural origin are generated. Anthropic points are either generated randomly across the geographic boundaries with the function RPG (Random Points Generator) or copied from a data frame that includes columns named x, y, year and Pnat. In the latter case, x and y are the points coordinates in a projected coordinate system; year must contain the same year numbers for which the point generation is performed and Pnat (probability of being of natural origin) must contain values between zero and one (values smaller than a threshold specified in the simulacro function will then be considered of anthropic origin). Natural points are generated in user defined, yearly numbers starting from extant points at that time step. Distances from existing points are sampled from a user defined One Dimensional Dispersal Kernel (function fx) and directions are sampled randomly. This part is done with the NPG (Natural Points Generator) function. Spatial filtering is done on both the natural and anthropogenic subsets through the function spatFilter, which deletes points from the input argument points based on the value of the raster’s cell (argument MAP) they fall into. In order to account for the reduction in the number of points after the filtering, an excess of points is initially generated (arguments FACNAT and FACANTH express factors by which the wanted number of points is multiplied before filtering). Eventual superfluous points are then randomly deleted to reach the user defined quantity.

###### Function modSel

modSel is used to compare several simulated time series with one observed invasion time series. It is a wrapper around the spatSim function which generates a measure of similarity between any two time series. This is done by computing Rippley’s K-function for the two datasets and measuring the sum of their square differences.

###### Inputs

Requested input files are of two types: invasion time series and spatial boundaries.

Invasion time series

The time series must be provided in the form of a data frame: one record per row containing the columns year, listing the year of the sighting in a numerical form; x and y, listing longitude and latitude respectively of the sighting locations in a projected coordinate system.

Spatial boundaries

The spatial boundaries must be in the form of either a 1) RasterLayer object with cell values of either 0 (indicating the area where the computation occurs) or >=1 (indicating the area outside the study system) or 2) a SpatialPoligon(s) object. Like for the time series and all other spatial information in the package, spatial boundaries must also be provided in a projected coordinate system. An example invasion time series can be called with the command:

data(frogs)

frogs contains year (year of the sighting), y (latitude in the New Zealand Transverse Mercatore coordinate system, length unit of measure is meter), x (longitude in the New Zealand Transverse Mercatore coordinate system, length unit of measure is meter). An example of spatial boundaries can be called with the command:

data(nzp)

nzp is a SpatialPolygons object with two features (the North and South islands of New Zealand).

### Sample analysis

Using the input data presented before, I show the typical Biolinv workflow. This allows to classify the sighting locations in a time series as either of anthropic or of natural origin, to quantify the human contribution to the invasion, to estimate the natural dispersal kernel, and to project the time series in the future.

###### Jackknife on invasion time series

data(frogs)

frogsJK<- jackKnife(DF= frogs, N= 10)

The first step of the analysis is optional but I suggest implementing the Jackknife approach especially in the case of small time series (few localities or few years). Jackknifing generates multiple datasets from the original time series, all of which will need to be processed in the same way. Ideally the processing can be done in parallel; naturally, the processing time increases considerably if Jackknifed datasets must be processed in series. All following instructions are supposed to be repeated for each of the Jackknifed datasets.

###### Running the EM algorithm

data(nzp)

data(frogs)

randp<- RPG(rpopn=1000, boundary=nzp, SP= 'random_frog')

frogsEM<- EM(dataset= frogs, randompoints= randp, sigma=6, pi=0.5)

# plot output

plotlacro(x= frogsEM, outline= nzp)

Function EM runs the EM algorithm. The dataset parameter is the time series (frogs). The output of the EM function is a copy of dataset with a new column, frogs$Pnat, which indicates the probability of that point being of natural origin. Argument randompoints can be generated with the function RPG (Random Points Generator), which is used to generate a Poisson point process within the spatial boundaries of the analysis. This dataset is used to compute the nearest neighbour distances used in the EM algorithm. rpopn is the number of desired points to be generated (1000 is a good number for most cases). SP is the name to be stored in the column species of the output.

The output of the EM function and its argument dataset can be conveniently plotted by the function plotlacro, where argument x is the data frame to be plotted and argument outline is the spatial boundaries for the analysis*.*

###### Setting up time series simulation

idst<- frogsEM[1:10,]

Cr<- frogsEM[-(1:10),]

yr<- unique(Cr$year)

nNoYear<- rep(NA,length(unique(Cr$year)))

hNoYear<- rep(NA,length(unique(Cr$year)))

for(i in 1:length(unique(Cr$year))){

# Cr for that year:

CrYear<- Cr[Cr$year==unique(Cr$year)[i],]

# natural points for that year:

nNoYear[i]<- nrow(CrYear[CrYear$Pnat>=.5,])

# human points for that year:

hNoYear[i]<- nrow(CrYear[CrYear$Pnat<.5,])

}

AV<- c(2,3,4.5,7.5,11,15,20,25) #alpha values

frogsLacro<- simulacro(INIDIST=idst,YEARS=yr,

BOUNDARY=nzp,NNAT=nNoYear,NANTH=hNoYear,

FACNAT=10,

A=AV,X=seq(.1,30,.1),

TRUEANTH=TRUE,TRUEDB=Cr,PROB=.5,

ITERATIONS=10,HSM=nzp)

The simulacro function generates multiple (or one) virtual datasets that have the same anthropic points as the input dataset (frogs) and the same number of natural points. These are generated with varying natural dispersal kernels. The simulated dataset that most resembles the frogs dataset will then be selected in the next paragraph with the modSel function. The simulacro function requires considerable processing power to generate all the required datasets. Therefore, I suggest performing an initial test run using a low number of Alpha values and replicates before running the full dataset generation process.

The simulacro function requires several arguments: INIDIST is the initial distribution from which every simulated dataset starts. In the example above, the first ten records of frogs have been used for this (idst). YEARS is a vector of unique year numbers used to correctly name the output simulated time series. BOUNDARY is a map of the geographic boundaries within which points are generated (same as in previous functions). NNAT is either a vector of (integer) numbers of natural points to be generated per year or the mean (integer) number of natural points to be generated each year. NANTH is the same but for the anthropic points. FACNAT and FACANTH are factors by which the number of natural and anthropic points respectively are multiplied before the HSM filtering. They are only used if HSM is a raster file and should be set to higher values the lower the proportion of suitable cells in HSM. A is a vector of unique alpha values used to make virtual datasets. In the example, eight values are used, this means that eight datasets per replicate will be created by the simulacro function. Similarly, C is a vector of unique C values, however it is not necessary to set it to anything else than the default value of 2 (CIT my methods paper). X is a vector of possible dispersal distances used for simulating natural dispersal. This set of distances will be sampled from the One Dimensional Dispersal Kernel function with C= 2 and Alpha= A. TRUEANTH can be either “true” or “false”. If “true”, TRUEDB will be used to generate anthropic points, if “false” anthropic points will be generated with the RPG function in the quantities specified in NANTH. The use of this latter option is discouraged at this stage of the process as it is mainly meant for future forecasts of the invasion process (see below). Using TRUEDB will generate better datasets for the estimation of the dispersal kernel. TRUEDB is a data frame containing points of anthropic origin; must be in the same format as INIDIST. Rows where TRUEDB$Pnat>PROB will be ignored. PROB is a threshold value over which values in TRUEDB time series are considered of natural origin. ITERATIONS is the number of datasets with the same Alpha (A) and C values. For a full analysis, I suggest a higher number of iterations than the one in the example above (this function has tested positively with 90 replicates). HSM is the habitat suitability map. It can be a RasterLayer object, giving the probability of successful establishment of new populations, or a SpatialPolygons object, giving the boundaries within which new points are generated.

The output of the simulacro function is a list of lists of dataframes, each of which is a simulated time series. If DIR=TRUE one folder will also be created for each Alpha and C combination containing all the replicates datasets set in ITERATION. if DIR=FALSE (default) the order in the list will follow the order of the C and Alpha values respectively as set in C and A.

###### Selecting best time series simulation

data(nzw)

frogsSum<- modSel(WIN= nzw, M0= frogsEM, M2= frogsLacro,

AV= c(2,3,4.5,7.5,11,15,20,25), RAD= seq(0,30000,1000))

# plot output:

plotAlpha(SSIM= frogsSum, REP= 10)

plotAlpha(SSIM= frogsSum, REP= 10, BP=TRUE)

To select the simulated time series that is the most similar to my observed one (frogs) the modSel function is used. It takes five arguments. WIN is the spatial boundaries of the analysis (like nzp) in the form of a “window” object from package “spatstat”. An example WIN object (nzw) is called in the first row. M0 is the output of the EM function (observed time series). M2 is the output of the simulacro function (simulated time series list). AV is a numeric vector of the Alpha values used in the simulated datasets in the same order as in the list of argument M2. It is used to save the output data frame. RAD is a numeric vector of search distances for the K-function. This parameter indicates the radiuses at which the K-function K(r) is evaluated. Fewer values speed up the computation time but produce less accurate results.

The output of this function is a data frame with two columns: dissimilarity contains the dissimilarity values computed by the spatSim function; compAlpha contains the input values from AV. This data frame can be conveniently visualised with the plotAlpha function. When argument BP is set as “true” all replicates are represented as boxplots, otherwise only the average line is plotted.

###### Future projections

idst<- frogsEM

yr<- seq(2015,2030,1)

hNoYear<-rep(c(1,0,0),length.out=length(yr))

AV<- 20

frogsLacro<- simulacro(INIDIST=idst,YEARS=yr,

BOUNDARY=nzp,NNAT=2,NANTH=hNoYear,

A=AV,X=seq(.1,30,.1),

TRUEANTH=FALSE,PROB=.5,

HSM=nzp,FACNAT=2)

f<- frogsLacro[[1]][[1]]

f<-f[f$year>2010,]

plot(nzp)

points(idst$x, idst$y,pch=3)

points(f[f$Pnat>=.5,'x'],f[f$Pnat>=.5,'y'],col=4)

points(f[f$Pnat<.5,'x'],f[f$Pnat<.5,'y'],col=2)

Function simulacro can also be used to project an invasion into the future. The main differences with respect to the use described above are the argument YEARS, that for future projections must contain the year’s numbers for which to produce new points; and the argument TRUEANTH, which must be set as =FALSE. The mean number of anthropic and natural points to be generated per year can be computed by averaging the number of anthropic and natural points per year as classified by function EM. Note that values of less than one are not acceptable as arguments NNAT and NANTH represent numbers of points to be generated.

### Conclusion and future implementations

Biolinv is a concrete step forward towards modelling and forecasting biological invasions, especially when accounting for anthropic dispersal.

Planned improvements in this package include the possibility of running the EM and simulacro functions on non-random anthropic release patterns, and allowing the HSM to change over time within the simulacro algorithm to account for climate change. The package is available on the CRAN website (https://cran.r-project.org/package=Biolinv).
