# Supplementary figures and images for: A new method for modelling biological invasions from early spread data accounting for anthropogenic dispersal
Source: PLoS One. 2018 Nov 27;13(11):e0205591. doi: 10.1371/journal.pone.0205591 (PMC6258513; doi:10.1371/journal.pone.0205591)

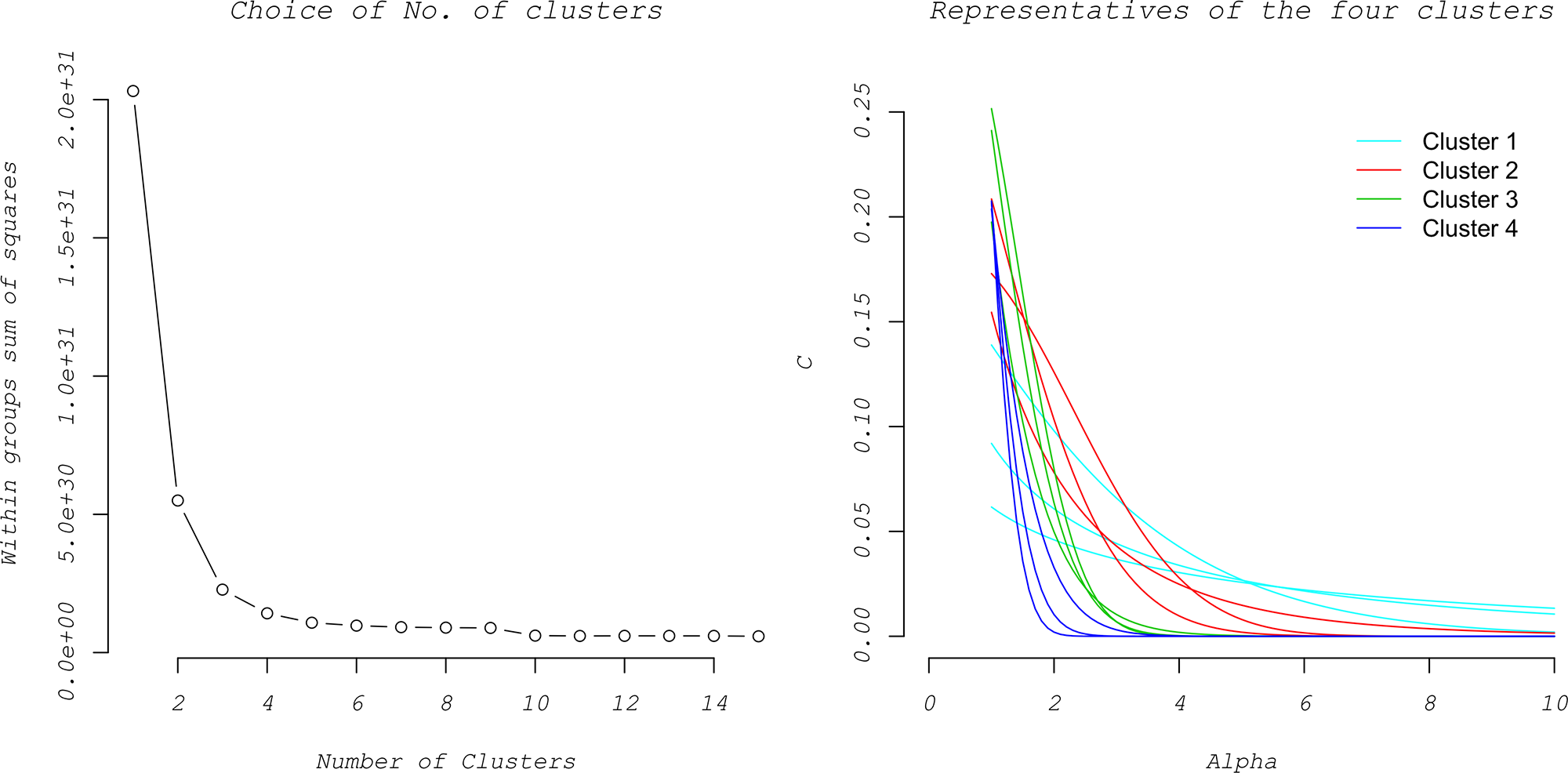

Supplement: S1 Fig — “Choce of No. of clusters”: Plot of the within-groups sum of squares against the number of groups for choosing the number of groups for the cluster analysis. “Representatives of the four clusters”: Few dispersal kernels grouped in their respective cluster. (PNG) [file pone.0205591.s003.png]

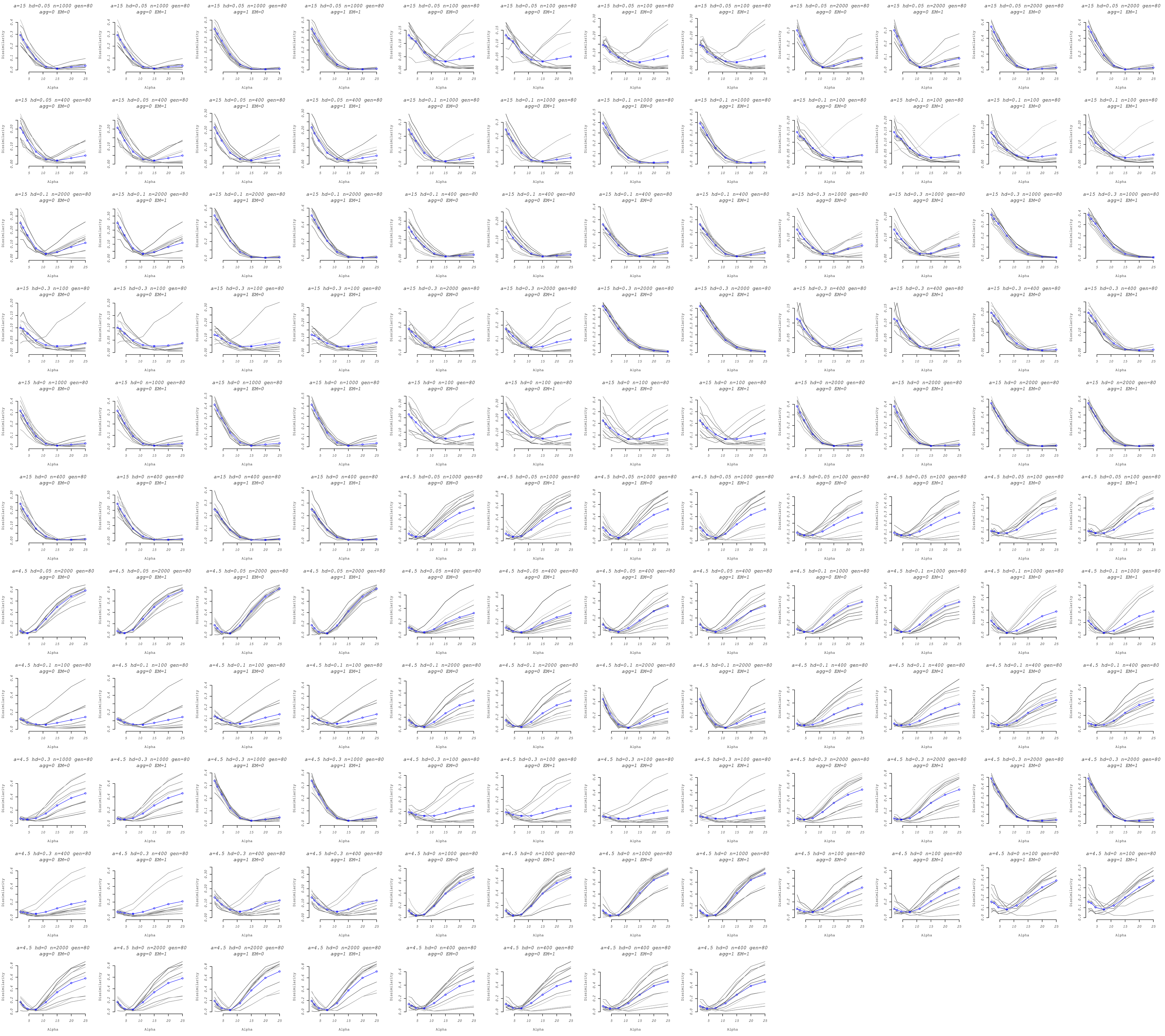

Supplement: S2 Fig — Dissimilarity curves of the α estimation of all 320 virtual datasets. (PNG) [file pone.0205591.s004.png]

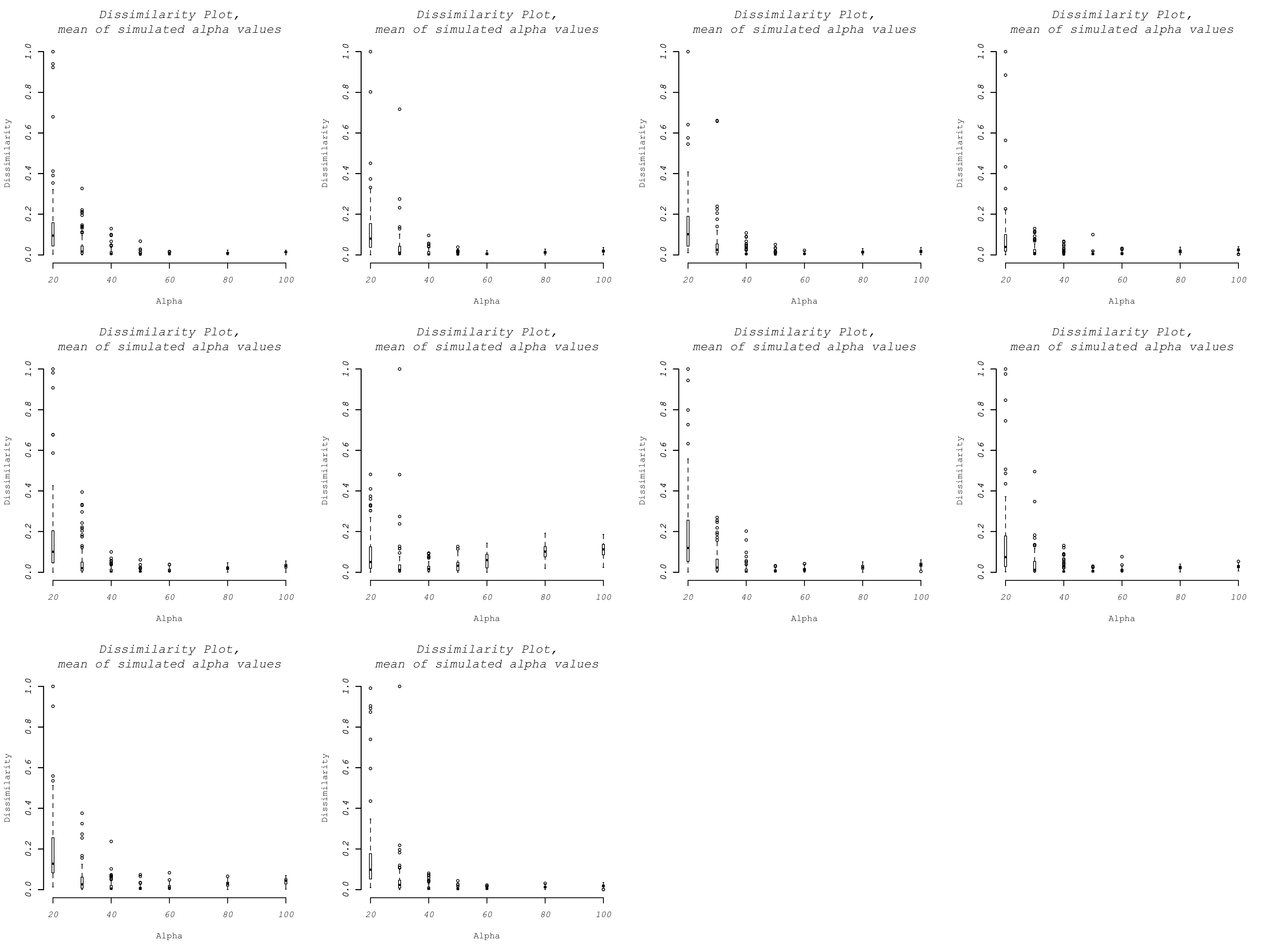

Supplement: S3 Fig — Dissimilarity values between comparison and observed datasets for the Litoria raniformis time series. Each boxplot represents similarity values of all simulated datasets with the same α value. Each plot is a Jackknifed dataset. HSM filtering was performed. (PNG) [file pone.0205591.s005.png]
